# The Impact of Financial Coaching on Health and Finances in Older Scam and ID Theft Victims

**DOI:** 10.1093/geroni/igaa057.989

**Published:** 2020-12-16

**Authors:** Peter Lichtenberg, LaToya Hall, Rebecca Campbell, Evan Gross

**Affiliations:** Wayne State University, Detroit, Michigan, United States

## Abstract

Population based and clinical samples consistently demonstrate that older financial exploitation (FE) victims are vulnerable across multiple domains. Our earlier research indicated that older scam and identity theft victims were vulnerable across multiple health and mental health dimensions as compared to a control group. The current work is a longitudinal study of 20 financial coaching participants and 20 controls. Program participants received individual financial coaching to help with credit problems, reporting and collections after they were scam or identity theft victims. Both groups received baseline assessment and 6 month followups (from the time of the end of the coaching). The mean age of the sample was 69 years, with a mean of 15 years of education. Seventy percent of the sample were women and African Americans. Control participants had fewer health conditions and better memory and executive functioning at baseline as compared to coaching participants. While there were no significant health measure changes in the control group, the coaching group demonstrated significantly decreased anxiety at follow up and trends for improved executive functioning and IADLs. Further, while financial stress and social support were significantly lower for the coaching group at baseline, at followup there were no group differences in these measures. The study results provide evidence that the financial coaching program has served as a protective factor in negating many of the adverse outcomes associated with FE and supports financial coaching programs as an appropriate intervention. In addition the coaching services saved or returned monies to nearly 65% of participants.

